# Commentary: Real-world post-deployment performance of a novel machine learning-based digital health technology for skin lesion assessment and suggestions for post-market surveillance

**DOI:** 10.3389/fmed.2024.1345659

**Published:** 2024-02-06

**Authors:** Alexander D. G. Anderson, Serigne N. Lo, Pascale Guitera

**Affiliations:** ^1^Royal Cornwall Hospital Trust, Truro, United Kingdom; ^2^University of Exeter, Exeter, United Kingdom; ^3^Melanoma Institute Australia, Sydney, NSW, Australia; ^4^The University of Sydney, Darlington, NSW, Australia; ^5^Sydney Melanoma Diagnostic Centre (SMDC), Camperdown, NSW, Australia

**Keywords:** artificial intelligence, skin cancer, AI for skin cancer, AI as a medical device, DERM, deep ensemble for the recognition of malignancy, Skin Analytics

## Introduction

The team at Skin Analytics are to be congratulated on developing the UK's first and only Class II licensed medical device incorporating AI for the diagnosis of skin cancers. They present their initial experience with real world cases in the above paper, which uses post market surveillance data relating to deployment in two NHS hospital trusts to assess the performance of the “DERM” algorithm. The algorithm was used as a tool for triaging dermoscopic images of skin lesions of concern referred by GP's on the UK “2 week wait” skin cancer pathway. Part way through the data collection period, the DERM algorithm was modified. Earlier patients were triaged using the “DERM-vA” algorithm and later patients with “DERM-vB.” Results for each version of the algorithm and each clinical site are reported separately.

A subset of all referrals was triaged by the AI algorithm. Cases flagged as possibly malignant were referred on to the NHS trust for dermatologist triage. Cases flagged as eligible for discharge were reviewed by a consultant dermatologist working for Skin Analytics and acting as a “second reader.” Based on this clinical assessment, these patients were either discharged or referred on to the NHS trust for further assessment.

## Analysis

Sensitivity relates to the likelihood of missing a skin cancer, which is of paramount importance in terms of clinical safety. The two algorithms are reported as having very high sensitivities (95–100%) in both clinical settings. In particular, zero skin cancers of any type were identified in a total of 1,783 patients (1,486 from UHB and 297 from WSFT) assessed as “eligible for discharge” by the modified DERM-vB algorithm. The authors go so far as to conclude that the performance “provides sufficient evidence to support the removal of the second-read for low-risk lesions in order to maximize health benefits safely.”

In order to provide a reliable estimate of sensitivity, one would expect data to relate to one or more consecutive series, with a full accounting for excluded patients and missing data (1). Nevertheless, in the current publication, only 10,925 out of 14,500 cases (75.3%) were assessed by DERM. A number of potential exclusion criteria and “technical issues” are cited as reasons for excluding the remaining 24.7%. Unfortunately, no further case breakdown is provided for this significant proportion of patients who weren't assessed by DERM. This introduces the possibility of potential selection bias, particularly if clinically ambiguous cases were excluded from analysis.

Cases “where the final diagnosis is still pending” were also excluded from some of the analysis, again without a detailed breakdown. In order to attempt to quantify this further, we have produced the summary table below, based on the data presented in Figure 3 of the original paper (Table 1). The summary table shows the prevalence of all skin cancers detected in the study population, with breakdown for each clinical site and for each of the 2 phases of the study. Given that the only difference described between the DERM-vA phase of the study and DERM-vB phase was a refinement in the algorithm, one would expect this proportion to remain fairly constant for each site across the 2 phases of the study: 1,833 skin cancers were detected in an overall population of 14,500 referrals, giving an overall prevalence of 12.6%. Of these, 1,006/1,833 (54.9%) were BCC. Prevalence appears low compared to previous UK series of 2ww skin cancer referrals, which report prevalence of MM or SCC alone in the region of 12% (2).

**Table 1:** Number of cases in which skin cancer was detected vs. total number of cases^*^.

	**UHB**	**WSFT**
DERM-vA phase	1,142/7,171 (15.9%)	173/1,119 (15.5%)
DERM-vB phase	382/4,800 (7.96%)	136/1,410 (9.65%)
*P*-value (Chi-square)	< 0.0001	< 0.0001

Data based on Figure 3 of the original paper.

^*^Includes MM, SCC, BCC, and rare skin cancers.

Furthermore, it can be seen that the prevalence of recorded skin cancers dropped by ~50% in UHB and almost 40% in WSFT in the DERM-vB phase of the study compared to the chronologically earlier DERM-vA phase. Whilst it is possible that a systematic change in referral population or criteria could have coincided with modifying the algorithm at a single site, the drop was replicated at both sites. This, together with the overall low prevalence, suggests to us that a large number of skin cancers present in the study population were not reported, particularly in the chronologically later phase of the study. It is possible that the current well-recognized pressures on NHS waiting lists resulted in a subset of patients being listed for excision or biopsy but awaiting a procedure or histological result at the time of data analysis. Patients in this position would have erroneously appeared as benign in the case level results reported in Figure 3 of the original paper and would have been excluded from assessment in the lesion-level population (attrition bias).

Finally, a cohort of lesions termed “atypical naevi” has been excluded from the analysis of benign lesions. Differentiating between malignant melanoma and benign naevi (atypical or otherwise) is one of the more demanding tasks in skin lesion diagnosis and excluding this cohort is likely to have resulted in inflated specificity estimates. For example, using DERM-vB at UHB, the reported specificity for benign lesions was 73.4%, but only 78/193 of “atypical naevi” were classified as non-malignant by the algorithm, giving a specificity of just 40.4% in this important subgroup.

## Discussion

Assessing the utility and safety of AI algorithms as potential skin cancer screening tools is an important healthcare priority. Unfortunately, the lack of transparency and failure to apply adequate scientific rigor to the current description of post-market implementation of the DERM algorithm do not support the use of AI alone as a screening tool. In particular, the reported sensitivity and missed cancer rates are potentially compromised by significant selection and attrition bias. Future descriptive studies should include consecutive series with full delineation of excluded cases and missing data and record histopathological assessment relating to appropriate cases across the entire population.

## Author contributions

AA: Writing—original draft, Writing—review & editing. SL: Formal analysis, Methodology, Writing—review & editing. PG: Conceptualization, Supervision, Writing—review & editing.
